# High-Performance Ppb Level NO_2_ Gas Sensor Based on Colloidal SnO_2_ Quantum Wires/Ti_3_C_2_T_x_ MXene Composite

**DOI:** 10.3390/nano12244464

**Published:** 2022-12-15

**Authors:** Baohui Zhang, Chong Li, Min Li, Chen Fu, Ran Tao, Honglang Li, Jingting Luo

**Affiliations:** 1 Key Laboratory of Optoelectronic Devices and Systems of Education Ministry and Guangdong Province, College of Physics and Optoelectronic Engineering, Shenzhen University, Shenzhen 518060, China; 2 College of Electrical Engineering, Nanjing Vocational University of Industry Technology, Nanjing 210023, China; 3 National Center of Nanoscience and Technology, Beijing 100190, China; 4 GBA Research Innovation Institute for Nanotechnology, Guangzhou 510535, China; 5 Guangdong Guangnaxin Technology Co., Ltd., Guangzhou 510535, China

**Keywords:** Ti_3_C_2_T_x_ MXene, SnO_2_, gas sensor, NO_2_, nanocomposite

## Abstract

Nitrogen dioxide is one origin of air pollution from fossil fuels with the potential to cause great harm to human health in low concentrations. Therefore, low-cost, low-power-consumption sensors for low-concentration NO_2_ detection are essential. Herein, heterojunction by SnO_2_ quantum wires, a traditional metal oxide NO_2_ sensing material, and Ti_3_C_2_T_x_ MXene, a novel type of 2D layered material, was synthesized using a simple solvothermal method for enhancing gas-sensing performance and reducing operating temperature. The operating temperature was reduced to 80 °C, with a best performance of 27.8 and a fast response and recovery time (11 s and 23 s, respectively). The SnO_2_ and Ti_3_C_2_T_x_ MXene composite exhibits high speed and low detection limit due to the construction of the heterojunction with high conductive Ti_3_C_2_T_x_ MXene. The selectivity and stability of gas sensors are carried out. This could enable the realization of fast response, high-sensitivity, and selective NO_2_ sensing under low operating temperatures.

## 1. Introduction

The development of urbanization, industrialization, and modern agriculture greatly facilitates human daily life, while greatly disturbing the ecological environment. Nitrogen dioxide (NO_2_) is a highly reactive and toxic gas generated from fossil fuels (heating, power, engines, chemical industry, etc.) [1,2]. It can lead to acid rain, and its salts are the main component of PM (particulate matter in the atmosphere) [3]. Even at low concentrations under 1 ppm, it can cause an increase in symptoms of bronchitis in asthmatic children and cause lung function damage due to its strong oxidizing properties [4,5,6]. In total, 92% of the global population lives in cities with air pollution exceeding limits from a WHO report [7], indicating the need for further monitoring and control of low-concentration air pollutant gases.

Gas sensors based on metal oxide semiconductors (MOS)—such as ZnO [8], SnO_2_ [9], WO_3_ [10], TiO_2_ [11], etc.—have played an important role in NO_2_ sensing due to their high sensitivity, fast response, and low cost. SnO_2_, as a typical n-type MOS, is one of the most sensitive materials due to its high absorption [12,13]. Most SnO_2_ sensors operate at a high temperature (300–400 °C) for better gas sensitivity, which not only reduces the sensor lifetime but also poses a risk of fire during long operation times [14,15,16,17]. At room temperature, the ultra-high resistance of SnO_2_ sensors results in low response to NO_2_ due to increased resistance as a result of surface reaction. Currently, improving gas adsorption and electronic transduction of MOS materials is a major problem in fabricating low-operating-temperature gas sensors. Recently, some researchers decreased the operating temperature of SnO_2_-based gas sensing materials by reducing dimensions and controlling the morphology of SnO_2_ [18,19,20,21]. For example, Zhong et al. [22] synthesized SnO_2−x_ nanosheets, showing a high response of 16 to 5 ppm NO_2_ at room temperature and taking more than 1000 s for recovery in 2019. In 2021, Hung et al. used SnO_2_ nanowires as sensing material. They had 50 and 100 s response and recovery times, respectively, under UV light [23]. In the same year, Zhou et al. made hollow SnO_2_ microspheres using colloidal nano SnO_2_ for NO_2_ sensing at room temperature, reaching a response of 10 to 10 ppm NO_2_ and a fast recovery time of 65 s [24].

Reducing dimensions and controlling the morphology is an active pathway for reducing the operating temperature of SnO_2_ gas sensors. The carrier transportation limited the electron injection for NO_2_ desorption and resistance reduction. Low-dimensional layered materials with high mobility were used for improving response speed at low operating temperatures. In 2015, Li et al. [25] used rGO mixed with SnO_2_ nanoparticles as a sensing material with fast response but slow recovery. Inaba et al. used SnO_2_-decorated SWCNT as a sensing material, reaching a response of 19 at 1 ppm NO_2_ under UV [26]. Ti_3_C_2_T_x_, the first-layered MXene which is synthesized via HF etching of Ti_3_AlC_2_ using MAX by Gogosti in 2011, with rich active surface groups and ultra-high conductivity, has shown great attraction for ammonia and VOC sensing in recent research [27,28,29,30]. It was also used as a composite, with SnO_2_ improving its gas sensing performance. Liu et al. made a SnO_2_/Ti_3_C_2_T_x_ composite with response close to 50 ppb NO_2_ at room temperature but full recovery at 100 °C [31]. Composites with 2D materials show potential for low-concentration and low-operating-temperature NO_2_ sensors for environmental monitoring.

Herein, we processed SnO_2_ quantum wires and Ti_3_C_2_T_x_ MXene composite for reducing operating temperature. Series mass of MXene was added for turning gas-sensing performance and operating temperature. Gas-sensing performance and characterizations were tested for explaining potential ppb-level NO_2_-sensing mechanism. It has been found that Ti_3_C_2_T_x_ MXene provided a highly conductive pathway for improved charge transportation, resulting in high- and fast-response SnO_2_/MXene composite NO_2_ sensors at low operating temperatures.

## 2. Materials and Methods

### 2.1. Materials and Synthesis

All the reagents were utilized as supplied without additional purification treatment. The Ti_3_AlC_2_ powder was purchased from Jilin 11th Technology Co. Ltd. (Jilin, China). Oleic acid (OA) and oleyl amine (OLA) were brought from Alfa Aesar. LiF and SnCl_4_·5H_2_O were from Shanghai Aladdin Biochemical Technology Co., Ltd. (Shanghai, China). HCl, toluene, and ethanol were from Sinopharm Chemical Reagent Co., Ltd. (Shanghai, China) The resistivity of the DI water employed throughout the whole experiment was around 18 MΩ·cm.

**Figure 1:**
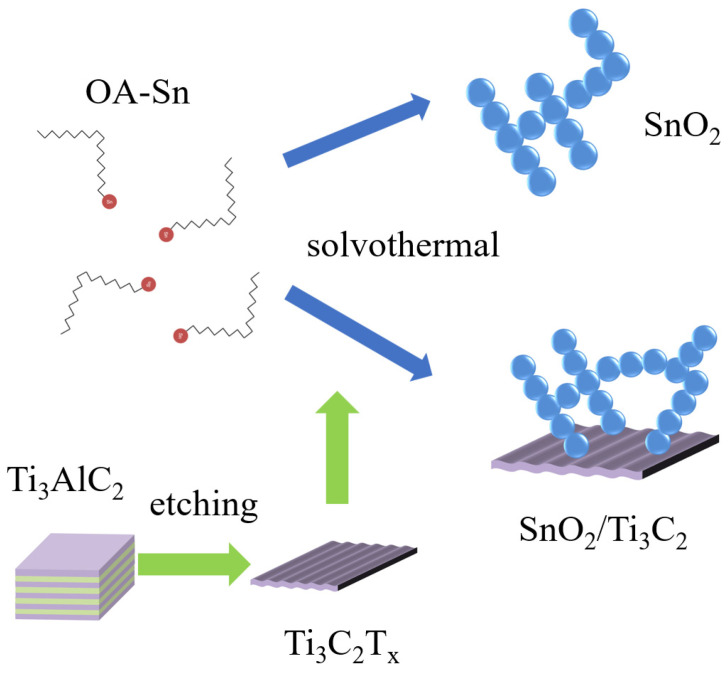
SnO_2_ and SnO_2_/Ti_3_C_2_T_x_ composite synthesis scheme.

All methods of synthesis and testing are shown in Figure 1. The pristine Ti_3_C_2_T_x_ MXene was prepared through the in situ HF generation etching method according to reports in the literature [27]. An amount of 1.65 g LiF was added to 15 mL HCl and 5 mL DI water mixture (forming 9M HCl at last) in a Teflon lining. 1 g of Ti_3_AlC_2_ powder was slowly added into the above solution in an ice bath, preventing the solution from overheating, and then magnetically stirred continuously for 24 h in a 40 °C oil bath. After etching, the product was washed and centrifuged repeatedly with DI water until the pH of the supernatant returned to 7. The Ti_3_C_2_T_x_ MXene was pumped, filtered, and dried at 60 °C under vacuum overnight, and powder was collected for composite.

The colloidal SnO_2_ was synthesized using a simple solvothermal method, shown in Figure 1 [32]. Firstly, 0.7 g SnCl_4_·5H_2_O (2 mmol) was mixed in 20 mL of oleic (OA) and 1.5 mL of oleyl amine (OLA). Then, the mixture was ultrasonically dispersed for 30 min until transparent. Next, the mixture was transferred into a Teflon-lined steel autoclave with 10 mL ethanol to react at 180 °C for 3 h. After rapid cooling to room temperature, the mixture was precipitated with ethanol and redispersed in toluene three times. Ti_3_C_2_T_x_ MXene was dissolved in ethanol (forming 3, 6, 9, 12 mg/mL for Moore ratios of 10%, 20%, 30%, and 40%, respectively, to SnO_2_) for composite synthesis. Finally, all the precipitate was dispersed in toluene for sensor fabrication. These materials and sensors with differently treated materials were marked as SnO_2_, ST3, ST6, ST9, and ST12, respectively.

### 2.2. Material Characterization

The crystal phase was analyzed with X-ray diffraction analysis (XRD; Rigaku D/Max 2550, Akishima, Japan) using Cu Kα radiation (λ = 1.5418 Å) in the 2θ range of 5−80°. The microstructure was observed via scanning electron microscopy (SEM; Sigma 300 Zeiss, Oberkochen, Germany) with an acceleration voltage of 15 kV. The element ratio and spot pattern scanning analysis were tested using energy dispersive X-ray spectroscopy (EDS) with SEM. Transmission electron microscopy (TEM; FEI Tecnai G2 F20 S-Twin, Hillsboro, OR, USA) was conducted with an acceleration voltage of 200 kV. X-ray photoelectron spectroscopy (XPS) data were obtained on a Thermo Fisher Scientific K-Alpha (Waltham, MA, USA) with an Al source, and the sample was prepared via drop casting Ti_3_C_2_T_x_ MXene on Si substrate.

### 2.3. Gas Sesnor Fabrication and Testing

Alumina ceramic plate with inter-digital Ag electrodes was used as the substrate of the gas sensor. Before film fabrication, devices were cleaned with acetone and absolute ethanol and then dried under nitrogen flow. The gas sensor was prepared via the spin-coating method. SnO_2_ and SnO_2_-MXene composite were dispersed in toluene, forming 20 mg/mL solution, were dropped onto the substrate, and then were spun at 2000 rpm for 30 s before being washed with methanol. These steps were repeated 3 times to form gas sensors. Then, the sensors were annealed at 300 °C for 3 h for surface OA removal.

The gas-sensing properties of the resistance sensor were evaluated using a Keithley 2400 digital source (Tektronix, Beaverton, OR, USA) meter with an 18 L chamber via the static sensing method. Temperature was raised with a heating plate and tested with a thermocouple. When the resistance of the sensors was stable, target gas with desired concentrations was injected into the chamber using syringes. As the sensor resistance reached a constant value, the chamber was opened for recovery in the atmosphere. The response of the gas sensor is defined as S = ΔR_g_/R_a_, where R_a_ is the resistance of the sensor in air (base resistance) and ΔR_g_ is the resistance change of the sensor in the target gas. The response time was defined as the time taken by the sensor to achieve 90% of the total resistance/frequency change in the case of gas adsorption. Similarly, the recovery time was defined as the time taken by the sensor response to reduce to 10% of its maximal value in the case of gas desorption.

## 3. Results

### 3.1. Materials Characterization

**Figure 2:**
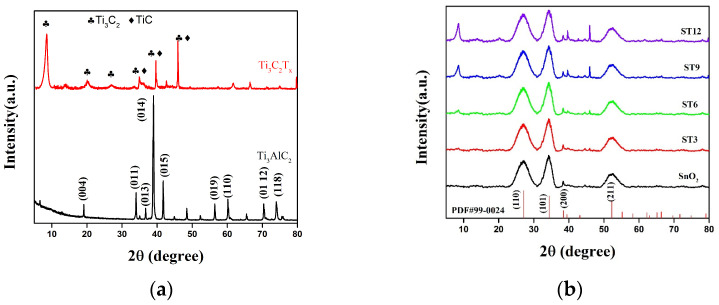
The X-ray diffraction patterns of (**a**) MAX and MXene, (**b**) SnO_2_, and their composites (ST3, ST6, ST9, and ST12).

Figure 2 displays the XRD patterns of MAX phase Ti_3_AlC_2_, Ti_3_C_2_T_x_ MXene, SnO_2_, and composite. Ti_3_AlC_2_ MAX powder shows intense crystalline peaks at 19.12°, 34°, 36.72°, 39°, 41.74°, 56.42°, 60.18°, 70.38°, and 74.06°, which are indexed to the (004), (011), (013), (014), (015), (109), (110), (01 12), and (118) diffraction planes, respectively, in Figure 2a [33,34]. The Ti_3_C_2_T_x_ peaks at 8.52°, 18.6°, and 28.7°indexed to the (002), (004), and (006) planes, respectively [27]. The peaks at 34.96°, 39.64°, and 45.96° were peaks of Ti_3_C_2_ and TiC which were over-etched. The reflection disappearance of highest (014) peak confirmed the Al was etched with HF. The small peaks between 35° and 45°correspond to the termination groups (–OH) and (−F), respectively, of MXene [35].

The XRD pattern of SnO_2_ showed reflection peaks at 26.58°, 33.86°, 33.94°, and 51.75°, corresponding to (110), (101), (200), and (211) plates, respectively, confirming its tetragonal rutile phase (JCPDS Card No #99-0024). The presence of the independent peaks of SnO_2_ and Ti_3_C_2_T_x_ MXene without any impurity peaks suggests the successful preparation of the composite (Figure 2b). The peak was almost invisible in the XRD pattern of ST3. Then, the peak intensity of Ti_3_C_2_T_x_ MXene significantly increases with an increase in its concentration in ST composite.

**Figure 3:**
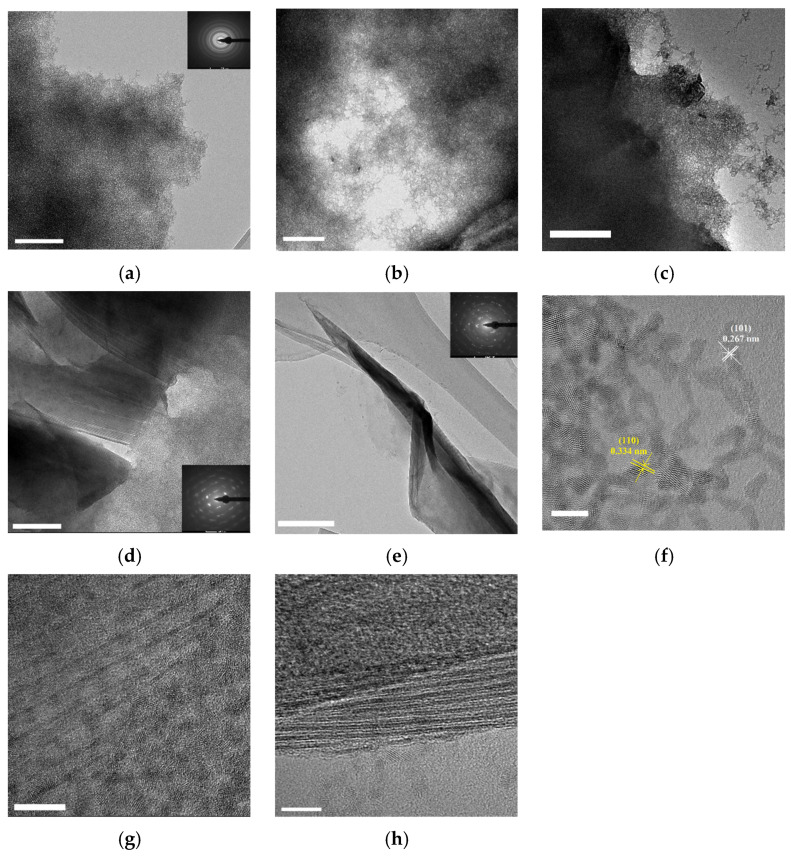
TEM of (**a**) colloidal SnO_2_, (**b**)ST3, (**c**) ST6, (**d**) ST9, (**e**) ST12 (scale bar 200 nm). High solution TEM of (**f**) SnO_2_, (**g**) ST9, and (**h**) ST12 (scale bar 5 nm).

Pure colloidal SnO_2_ was shown in Figure 3a. It shows that colloidal SnO_2_ was 2–3 nm diameter quantum dot necking in colloidal SnO_2_ networks. With the addition of Ti_3_C_2_T_x_ MXene (Figure 3b–e), colloidal SnO_2_ networks saw epitaxial growth on the surface of Ti_3_C_2_T_x_ MXene. The Ti_3_C_2_T_x_ MXene sheets were not visible in ST3 composite. With an increase in Ti_3_C_2_T_x_ MXene, the Ti_3_C_2_T_x_ MXene sheets were clearer, and colloidal SnO_2_ networks decreased on the Ti_3_C_2_T_x_ MXene. SAED (selected area electron diffraction) of SnO_2_, ST9, and ST12 is shown in the corresponding graph. Furthermore, the lattice spacings were found to be 0.334 and 0.267 nm, which are consistent with the (110) and (101) planes of rutile SnO_2_ [32], respectively (Figure 3f). Figure 3g shows the interface of Ti_3_C_2_T_x_ MXene and colloidal SnO_2_ networks of ST9; the MXene surface displayed fully epitaxial growth with SnO_2_. The interface of ST12 in Figure 3h shows that the surface was not full filled with SnO_2_.

**Figure 4:**
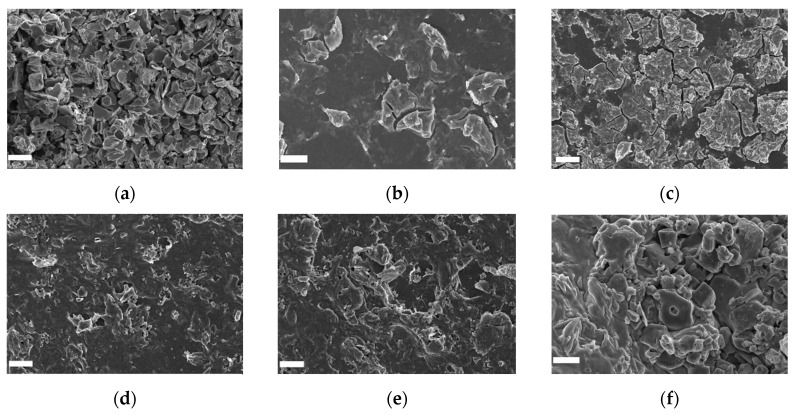
Film morphology of (**a**) Ti_3_C_2_T_x_ MXene, (**b**) SnO_2_, (**c**) ST3, (**d**) ST6, (**e**) ST9, and (**f**) ST12 by SEM (scale bar 10 μm).

The morphology of the as-prepared samples has been analyzed through employing SEM. As plotted in Figure 4a, the Ti_3_C_2_T_x_ MXene shows organ-like stack sheets after the HF etching. Figure 4b shows the morphology of pure SnO_2_ film. It shows a smooth colloidal film with some small stacks. ST3 film in Figure 4c has more stacks on the film surface for Ti_3_C_2_T_x_ MXene adding. With an increase in Ti_3_C_2_T_x_ MXene, the film turns rough. And the ST12 film shows sheets more like the Ti_3_C_2_T_x_ MXene film (Figure 4f).

**Figure 5:**
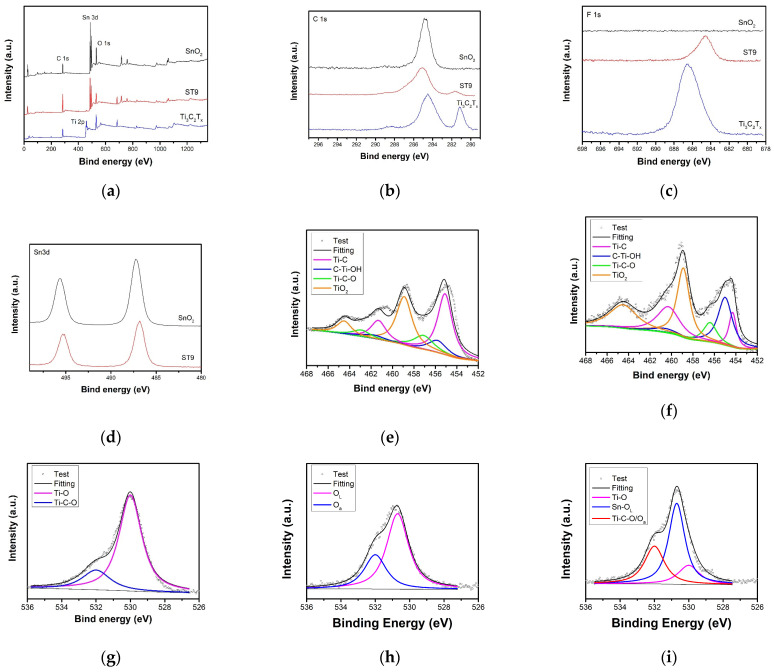
(**a**) XPS survey and high-resolution spectra of SnO_2_, Ti_3_c_2_T_x_ MXene, and ST9 composite. High-solution XPS spectra of (**b**) C 1s, (**c**) F 1s, and (**d**) Sn 3d. Ti 2p spectra of (**e**) Ti_3_C_2_T_x_ MXene and (**f**) ST9. O 1s spectra of (**g**) SnO_2_, (**h**) Ti_3_C_2_T_x_ MXene, and (**i**) ST9.

Figure 5 shows the XPS survey and high-resolution spectra of colloidal SnO_2_, Ti_3_C_2_T_x_ MXene, and ST9 composites. Colloidal SnO_2_ shows C–C bonds at 281.2 eV from surface OA ligand (Figure 5b). The Ti_3_C_2_T_x_ MXene shows two similar-intensity peaks at 281.2 and 284.2 eV ascribed to Ti–C and C–C bonds [36,37], respectively. The ST9 composite shows a weak Ti–C bond, and OA ligand was at the composite surface, introducing more C–C groups. F 1s high-resolution spectra with a binding energy of 686 eV was shown in Figure 5c. After mixture, the F 1s peak becomes weaker and redshifts. As shown in Figure 5d, the presence of two split peaks (Sn 3d5/2 and Sn 3d3/2 at 487.1 and 495.6 eV, respectively) confirms the formation of SnO_2_. The Sn peaks of ST9 are also weaker and slightly less redshifted than those of pure SnO_2_. The four pairs of peaks of Ti 2p centralized at 454.9 and 461.1, 455.8 and 461.4, 457.1 and 462.9, and 458.9 and 464.5 eV correspond to Ti–C, C–Ti–OH, C–Ti–O, and TiO_2_, respectively [38,39]. TiO_2_ and Ti–O groups strengthen the other active functional surface Ti groups against oxidation while using the solvothermal method (Figure 5e,f). The O 1s peaks at 530.7 and 532.5 eV can be assigned to the lattice O (O_L_, Sn–O–Sn) and chemically absorbed O (O_a_), respectively (Figure 5g). Figure 5h shows the O 1s peaks of Ti_3_C_2_T_x_ MXene at 529.9 and 532.2 eV for Ti–O and Ti–C–O groups, respectively [40,41]. In composite, the lattice O of SnO_2_ was the main oxide state and absorbed and Ti–C–O followed. There were still Ti–O groups on the composite.

### 3.2. Gas-Sensing Performance

**Figure 6:**
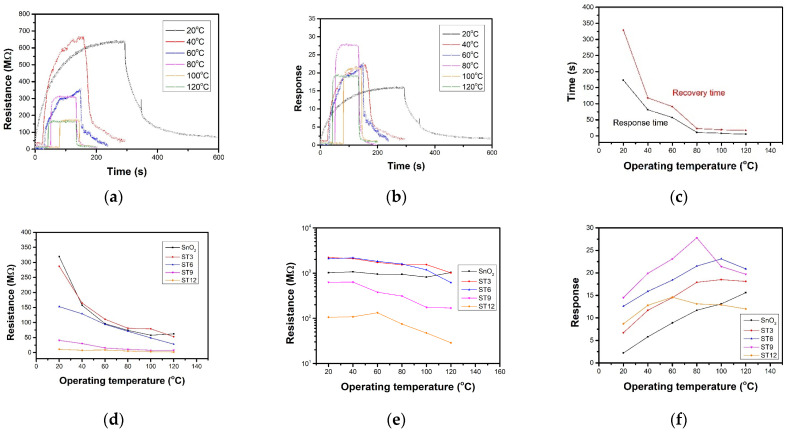
(**a**) Resistance and (**b**) response curves of ST9 sensor under different operating temperatures. (**c**) Response and recovery times of ST9 sensor under different operating temperatures. Target gas was 10 ppm NO_2_. (**d**) R_a_, (**e**) R_g_, and (**f**) responses of gas sensors under different operating temperatures.

Figure 6a displays resistance curves of ST9 sensor towards 10 ppm NO_2_ under series operating temperature. The resistance increases after exposure to NO_2_ for SnO_2_ as a p-type semiconductor. With the operating temperature increasing, the baseline resistance decreases from 40.9 MΩ to 8.1 MΩ due to high carrier transportation under high temperature. The resistance under target gas decreases, but the response shows an increasing trend at first below 80 °C and a decreasing trend as operating temperature increases from 15.6 to 27.8 in the calculated response curves in Figure 6b. Figure 6c compares the response and recovery time of ST9 sensor towards 10 ppm NO_2_ under different operating temperature. As operating temperature increases, the response and recovery changes quickly from over 100 s to around 10 s. The relationship of operating temperature to R_a_ and R_g_ and response towards 10 ppm NO_2_ was calculated and summarized in Figure 6d–f, respectively. It shows that as the operating temperature and the MXene ratio increase, gas sensor resistance decreases. As the Ti_3_C_2_T_x_ MXene increases, the optimal working temperature decreases. The response of pure SnO_2_ sensors still increases when the operating temperature increases up to 120 °C. The ST3 and ST6 sensor shows a best performance at 100 °C. ST9 and ST12 sensors shows the highest responses at 80 and 60 °C, respectively. The 2D Ti_3_C_2_T_x_ MXene sheets introduce an effective method of decreasing operating temperature.

**Figure 7:**
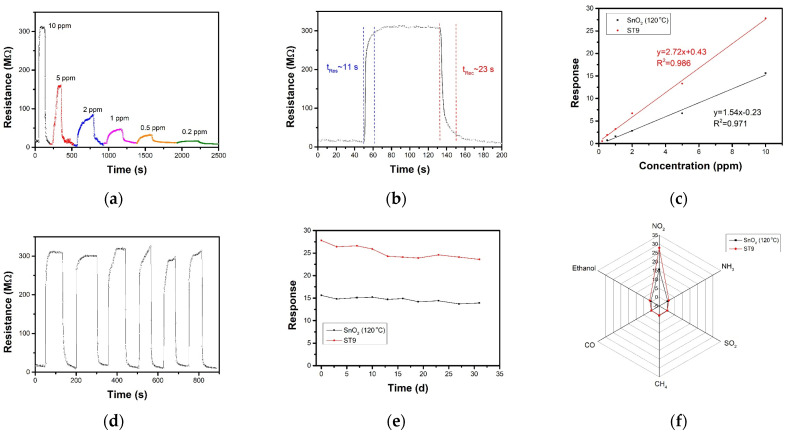
(**a**) Resistance curve of ST9 sensor towards series concentration NO_2_ at 80 °C. (**b**) Dependence of sensor response and the linear fitting upon NO_2_ concentration of ST9 sensor at 80 °C and SnO_2_ sensor at 120 °C. (**c**) Response and recovery time of ST9 sensor towards 10 ppm NO_2_ at 80 °C; (**d**) repeat curves of ST9 sensor towards 10 ppm NO_2_ at 80 °C. (**e**) Stability in a month of SnO_2_ and ST9 sensors towards 10 ppm NO_2_. (**f**) Selectivity of SnO_2_ and ST9 sensors toward 10 ppm NO_2_, NH_3_, SO_2_, CH_4_, CO, and ethanol.

Figure 7a presents the resistance curves of ST9 sensor under 80 °C towards series NO_2_ (10 ppm to 0.2 ppm). As the concentration decreases, the response becomes smaller and the response speed slower. The ST9 sensor showed the highest response of 27.8 toward 10 ppm NO_2_ at 80 °C, with response and recovery times of 11 and 23 s, respectively (Figure 7b). The NO_2_-sensing performance compared to that of other research was listed in Table 1. Figure 7c exhibits the linear fitting curve of the pure SnO_2_ and ST9 sensor response to NO_2_ concentration. The theoretical detection limits of the sensors were estimated to be 20 ppb (ST9) and 100 ppb (SnO_2_) according to the least-squares method. Repeatability and long-term stability are also two important aspects in gas-sensing applications. Figure 7d shows the repeat curves of ST9 sensor to 10 ppm NO_2_ 80 °C for 6 cycles, indicating a highly stable sensing performance. Furthermore, the long-term responses for a month of pure SnO_2_ and ST9 sensor to 10 ppm NO_2_ show a slight reduction under 10% (Figure 7e), indicating good long-term stability. The responses of pure SnO_2_ and ST9 sensor to 10 ppm NO_2_, CO, NH_3,_ SO_2_, CH_4_ and ethanol were shown in Figure 7f. The response towards NO_2_ was much higher than the other gases, indicating that the SnO_2_ and ST9 had high selectivity to NO_2_, and selectivity of ST9 also improved to pure SnO_2_.

### 3.3. Gas-Sensing Mechanism

Based on its gas-sensing performance and material characterizations, the gas-sensing mechanism of SnO_2_/Ti_3_C_2_T_x_ MXene composite has been hypothesized. The SnO_2_ sensing mechanism is traditionally analyzed using the surface control model [47], which is based on the interaction between chemisorbed oxygen species and target gases on the surface of SnO_2_. Generally, oxygen in ambient air adsorbed on metal oxide surfaces converted to O_2_^–^, O^–^, and O^2–^ by capturing electrons near the valence band. In our work, O_2_^–^ and O^–^ were the main absorbed oxygen states on the SnO_2_ surface following the reaction [47]:

(1) $$O_{2}+e^{-}\to O_{2}^{-}$$

(2) $$O_{2}^{-}+e^{-}\to 2O^{-}$$

**Figure 8:**
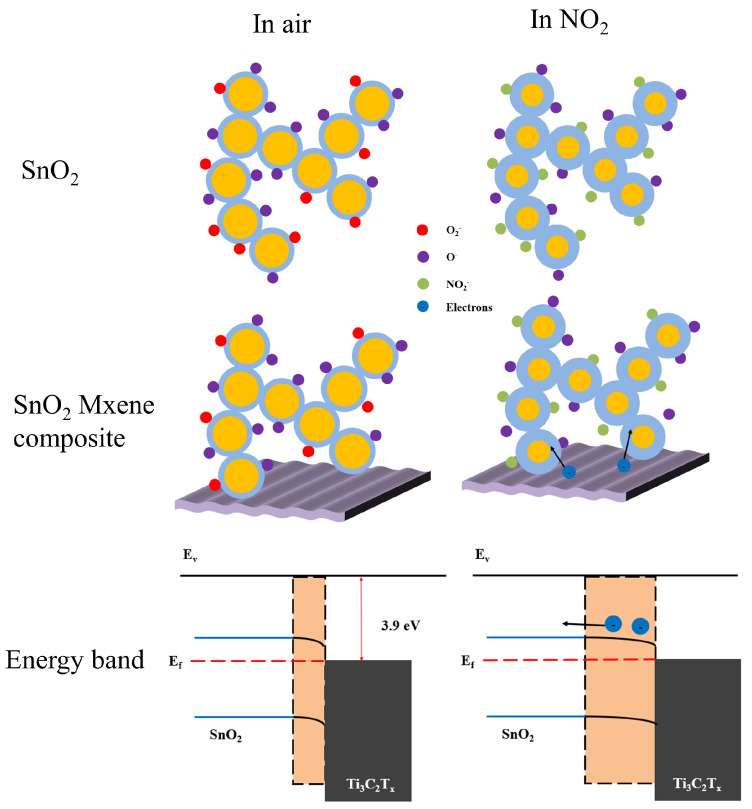
Schematic illustration of NO_2_-sensing mechanism for SnO_2_ and SnO_2_-Ti_3_C_2_T_x_ composite sensor.

Normally, the O^2−^ was mostly formed above 400 °C. The surface-absorbed oxygen formed a depletion zone on the SnO_2_ surface (blue area in Figure 8). This caused the carrier balance to begin forming the resistance of gas sensors. When exposed to NO_2_, the surface-absorbed oxygen reacts with NO_2_ per follow equation [48]:

(3) $$O_{2}^{-}+{\mathrm{NO}}_{2}+e^{-}\to {\mathrm{NO}}_{3}^{-}+O^{-}$$

This reaction introduces the consumption of electronics forming a deeper depletion zone on the SnO_2_ surface, leading the resistance of gas sensor increase.

With the addition of Ti_3_C_2_T_x_ MXene, SnO_2_/Ti_3_C_2_T_x_ MXene heterostructures are formed with Schottky barriers. The Fermi level of SnO_2_ was at 4.5 eV [49], higher than the Ti_3_C_2_T_x_ MXene work function (~3.9 eV) [50]. The Fermi level balance leading electron injecting from the MXene to SnO_2_ and formed the band bending. Another electron depletion region between the interface of SnO_2_ and Ti_3_C_2_T_x_ MXene was formed. When exposed to NO_2_, two depletion regions were both widened for electron consumption, enhancing NO_2_ sensing performance. The Ti_3_C_2_T_x_ MXene sheet introduced folded and stacked multilayer into the film, which offered a large number of active sites and gas molecule transport channels for the adsorption of oxygen and NO_2_, thus improving the NO_2_-sensing response. In addition, the high conductivity of Ti_3_C_2_T_x_ MXene formed a carrier transportation channel during the sensing process and decreased the response and recovery time. The performances of ST3 and ST6 sensors were improved, but the Ti_3_C_2_T_x_ MXene was not enough for carrier transportation. The ST12 sensor response decrease was due to the Ti_3_C_2_T_x_ MXene surface not being fully grown, transporting more electrons for band balance and reflecting Ti_3_C_2_T_x_ MXene p-type response to gases, opposite to the n-type SnO_2_.

## 4. Conclusions

In summary, SnO_2_ quantum wires and Ti_3_C_2_T_x_ MXene composites with different ratios were synthesized using the one-step solvothermal method. SnO_2_ quantum wire epitaxial growth occurred on the surface of Ti_3_C_2_T_x_ MXene. Ti_3_C_2_T_x_ MXene was oxidized during the solvothermal method. The molar ratio was 30% (MXene); the marked ST9 sensor showed the best response of 27.8 to 10 ppm NO_2_ at 80 °C, with 11 s and 23 s of response and recovery time, respectively. The response of ST9 sensor was 2 time to pure SnO_2_ at an operating temperature of 120 °C. The ST9 sensor could detect NO_2_ as low as 20 ppb in theory with excellent selectivity. The potential gas-sensing mechanism of SnO_2_/Ti_3_C_2_T_x_ MXene composite has been hypothesized to be the heterostructure enhancing the carrier transfer into SnO_2_, enhancing surface reaction and sufficient carrier supplied by the high conductivity of Ti_3_C_2_T_x_ MXene.

## Figures and Tables

**Table 1:** NO_2_-gas-sensing performance of SnO_2_-based sensors.

Materials	Response	Operating Temperature (°C)	Response/Recovery Time	LoD	Reference
SnO_2−x_ nanosheets	16@5 ppm	RT	331 s/1057 s	-	[22]
SnO_2_ nanowires	50@5 ppm	RT(UV)	420 s/100 s	100 ppb	[23]
SnO_2_ hollow microspheres	10@10 ppm	RT	17 s/65 s	26 ppb	[24]
Bio-templated SnO_2_ channels	35.9@100 ppm	RT	2.67 s/13 s	10 ppb	[42]
leaf-like SnO_2_	7@0.5 ppm	65	10 min/46 min	-	[43]
rGO/SnO_2_	84.5%@0.5 ppm	120	22 s/125 s	-	[44]
CNT/SnO_2_	0.59@0.5 ppm	RT	~40 s/~200 s	100 ppb	[45]
SWCNT/SnO_2_ nanoparticles	18@1 ppm	RT(UV)	200 s/-	1 ppb	[26]
MoS_2_/SnO_2_	34@100 ppm	RT	2.2 s/11 s	10 ppb	[46]
Ti_3_C_2_/SnO_2_	1.57@10 ppm	RT	~300 s/~100 s (100 °C)	50 ppb	[29]
Ti_3_C_2_T_x_/SnO_2_	27.8@10 ppm	80	11 s/23 s	20 ppb	This work

## Data Availability

All data, models, and codes generated or used during the study appear in the submitted article.
